# Plasma levels of monokine induced by interferon-gamma/chemokine (C-X-X motif) ligand 9, thymus and activation-regulated chemokine/chemokine (C-C motif) ligand 17 in children with Kawasaki disease

**DOI:** 10.1186/s12887-015-0424-6

**Published:** 2015-09-03

**Authors:** Siqi Feng, Shiv Kumar Yadav, Fang Gao, Qijian Yi

**Affiliations:** Department of Cardiovascular Medicine, Children’s Hospital of Chongqing Medical University, Chongqing, 400014 PR China; Ministry of Education Key Laboratory of Child Development and Disorders; Key Laboratory of Pediatrics in Chongqing, CSTC2009CA5002, Chongqing International Science and Technology Cooperation Center for Child Development and Disorders, Chongqing, 400014 PR China; Present address: Children’s Hospital of Chongqing Medical University, Zhongshan Er Road, No.136, Yuzhong District, Chongqing, 400014 PR China

**Keywords:** Kawasaki disease, Chemokine, T cell, Children

## Abstract

**Background:**

Monokines induced by interferon-gamma/Chemokine (C-X-C motif) ligand 9 (MIG/CXCL9), thymus and activation-regulated chemokine/Chemokine (C-C motif) ligand 17 (TARC/CCL17) are chemotactic factors that specifically collect and activate leukocytes, which are considered as chemoattractants of T helper cells. In the present study, we have investigated the effects of T helper type-1 (Th1) cells and T helper type-2 (Th2) cells in Kawasaki disease (KD) by determining plasma levels of MIG/CXCL9 and TARC/CCL17 and exploring the relationship between MIG/CXCL9, TARC/CCL17 levels and coronary artery lesions (CAL).

**Methods:**

Forty-three children patients with KD and 19 healthy controls were included in this study. General characteristics were obtained from all subjects. Plasma concentrations of chemotactic factors of MIG/CXCL9 and TARC/CCL17 were measured by enzyme-linked immunosorbent assay (ELISA) for all subjects.

**Results:**

Plasma levels of MIG/CXCL9, TARC/CCL17, and the ratios of MIG/TARC were significantly elevated in pediatric patients with KD compared to those in the control group. There were also significantly higher levels of MIG/CXCL9, TARC/CCL17, MIG/TARC ratios and prominently lower hemoglobin (Hb) levels in KD with CAL compared to KD without coronary artery lesions (NCAL). Hb was significantly decreased and plasma MIG/CXCL9 levels had a significantly negative correlation with CRP in KD with CAL patients (KD-CAL), whereas a positive correlation of plasma MIG/CXCL 9 with WBC was observed in KD without CAL patients (KD-NCAL).

**Conclusion:**

Th1 and Th2 cells may be involved in an imbalanced activation in pediatric KD patients during an acute period of the disease. Furthermore, immune lesions of vessels in KD patients may be mediated by the imbalanced activation of Th1 and Th2 cells.

## Background

Kawasaki disease (KD), also called mucocutaneous lymphnode syndrome (MCLS), was firstly described in 1967 [1]. It is a kind of self-limited systemic vasculitis characterized by a constellation of clinical signs and symptoms. The target vessels are small and medium-sized arteries, especially coronary arteries. Studies suggested that 15 %–25 % children with KD resulted in coronary artery lesions, if they wouldn’t get timely treatment [2]. KD has become the leading cause of acquired heart disease in children in developed countries.

The pathogenesis of KD has not been clearly identified yet. Many studies have revealed that super-antigens of infections could trigger the immunoreaction in KD resulting in the changes of a series of inflammatory factors, such as white blood cells (WBC), C-reactive protein (CRP) and erythrocyte sedimentation rate (ESR). T cells activation has been reported to be participated in the immunological pathogenesis [3, 4], and some pro-inflammatory cytokines of T cells, such as interleukin-4 (IL-4), interferon-gamma (IFN-γ), were changed in acute KD. However, little is known in KD patients about the chemotactic factors of T helper type-1 cells (Th1) and T helper type-2 cells (Th2), plasma monokines induced by interferon-gamma/Chemokine (C-X-C motif) ligand 9 (MIG/CXCL9) and thymus and activation-regulated chemokine/Chemokine (C-C motif) ligand 17 (TARC/CCL17). Lee CP et al. found that plasma TARC/CCL17 levels were higher in KD before immuoglobulin (IVIG) treatment compared to those in controls. However, the plasma TARC/CCL17 levels decreased significantly in KD after IVIG treatment [5]. No data about plasma MIG/CXCL9 levels have been reported so far, and little is known about the relationship between plasma MIG/CXCL9, TARC/CCL17 levels and coronary artery lesions (CAL) in KD patients. In the present study, we have measured plasma concentrations of MIG/CXCL9 and TARC/CCL17 in KD patients to reveal whether these chemotactic factors are involved in the function of T helper cells, and whether the balance between Th1 cells and Th2 cells is altered in KD patients.

## Methods

### Subjects and data collection

We have enrolled KD patients before intravenous immunoglobulin therapy and healthy children as controls at Children’s Hospital of Chongqing Medical University, Chongqing, P.R. China. The study groups included 43 KD patients (22 males and 21 females, average age 2.98 ± 1.91 years) and 19 healthy controls (12 males and 7 females, average age 3.37 ± 3.02 years). All of the KD patients were identified according to the criteria proposed by the Japanese Circulation Society Joint Working Group [6]. These KD patients were confirmed without any other associated immunological diseases. The study was approved by the Ethics Committee of Children’s Hospital, Chongqing Medical University, and written informed consents were obtained from the parents of all subjects.

The KD patients had echocardiography within 2 weeks from the onset. The echocardiographic diagnosis met the criteria proposed by the Japanese Kawasaki Disease Research Committee [7]. According to echocardiography parameters, 43 KD patients were divided into two groups, KD without coronary artery lesions (KD-NCAL) group (*n* = 21) and KD with coronary artery lesions (KD-CAL) group (*n* = 22). As we enrolled the study subjects in only 3 month, our coronary artery lesions morbidity rate was not consistent with the annual rate reported before but higher than that. These KD children were treated with 2 g/kg intravenous immunoglobulin and oral aspirin after admission.

White blood cells (WBC) counts, platelets (Plt) counts, percentage of neutrophils (N %), percentage of leukomonocytes (L %), hemoglobin (Hb), hematocrit (Hct), C-reactive protein (CRP), erythrocyte sedimentation rate (ESR), aspartate aminotransferase (AST), creatine kinase-MB (CK-MB) were obtained from all KD subjects; blood samples were drawn before intravenous immunoglobulin administration.

### Sample collection and processing

Venous blood samples were collected from KD patients before intravenous immunoglobulin administration on the day of admission using an ethylene diamine tetraacetic acid (EDTA) tube. And then the blood samples were centrifuged to separate plasma immediately at speed of 3000 revolutions/min for 5 min. The same proceeds have been done to obtain plasma samples from the healthy controls. All of the plasma samples were stored at –80 °C centigrade.

### Measurement of plasma MIG/CXCL9 and TARC/CCL17 levels

Plasma levels of MIG/CXCL9 and TARC/CCL17 in KD patients and healthy controls were determined by commercial enzyme-linked immunosorbent assay (ELISA) kits (Shanghai Hushang Biotechnology Co., LTD, Shanghai, China). The procedures were done following the manufacturers’ instructions. MIG/CXCL9 and TARC/CCL17 standards were run on micro-test plates, and the antigen concentrations (MIG/CXCL9: ng/L, TARC/CCL17: pg/ml) were determined from the standard curves by the micro-plate reader (Bioteck Epoch, US). All of the samples were measured in duplicate.

### Statistical analysis

Plasma levels of MIG/CXCL9 and TARC/CCL17 were analyzed as quantitative traits. All of the values are described as mean ± standard deviation (SD) or number and percent (*n*, %). Comparisons of frequencies between groups were analyzed using *χ*^2^ text. Differences between groups were assessed using Student’s *t*-text. Pearson’s correlation was used for associations between sequential parameters. The program SPSS19.0 for windows was used for all statistical analyses. All *P* values had 2-sides, where the *P* value < 0.05 was considered as statistically significant.

## Results

### Levels of MIG/CXCL9, TARC/CCL17 and ratios of MIG/TARC in study subjects

There were no significant differences between KD patients and healthy controls in age and sex (*P* > 0.05); Compared with healthy controls, plasma levels of MIG/CXCL9 (1110.16 ± 609.91 vs. 274.67 ± 142.44 ng/L, respectively), TARC/CCL17 (720.08 ± 257.63 vs. 341.99 ± 41.62 pg/ml, respectively) and the ratios of MIG/TARC (1.44 ± 0.42 vs. 0.74 ± 0.11) were significantly increased in patients with KD (Table 1).

**Table 1:** Plasma levels of MIG/CXCL9, TARC/CL17 and MIG/TARC in all study subjects

	KD (*n* = 43)	Controls (*n* = 19)	*P* value
Age at diagnosis(Yr)	2.98 ± 1.91	3.37 ± 3.02	0.57
Sex(male/female)	22/21	12/7	0.16
MIG/CXCL9(ng/L)	1110.16 ± 609.91	274.67 ± 142.44	<0.01
TARC/CCL17 (pg/ml)	720.08 ± 257.63	341.99 ± 41.62	<0.01
MIG/TARC	1.44 ± 0.42	0.74 ± 0.11	<0.01

*KD* Kawasaki disease, *Yr* year, *MIG/CXCL9* Monokine induced by interferon-gamma, *TARC/CCL17* Thymus and activation-regulated chemokine, *MIG/TARC* the ratio of plasma MIG/CXCL9 and TARC/CCL17 levels; Data are presented as Mean ± SD. *P*-value is for the comparison between controls and KD patients

### General characteristics and plasma MIG/CXCL9 and TARC/CCL17 levels, and ratios of MIG/TARC in KD-CAL and KD-NCAL

On the comparison between KD-CAL and KD-NCAL groups, there were no significant differences in age, sex, WBC, Plt, N %, L %, Hct, CRP, ESR, AST, CK-MB (*P* > 0.05). However, a significantly decrease in hemoglobin and an increase in plasma MIG/CXCL9, TARC/CCL17, and ratios of MIG/TARC were observed in KD-CAL group compared to KD-NCAL group (Table 2).

**Table 2:** General characteristics and plasma MIG/CXCL9 and TARC/CCL17 and MIG/TARC between KD with CAL (KD-CAL) and KD without CAL (KD-NCAL)

	KD-CAL (*n* = 22)	KD-NCAL (*n* = 21)	*P* value
Age at diagnosis(Yr)	2.96 ± 2.04	3.06 ± 1.84	0.95
Sex (male/female)	15/7	7/14	0.16
WBC (10^3^/ul)	14.31 ± 6.14	14.82 ± 4.96	0.73
Plt (10^3^/ul)	354.91 ± 99.62	378.33 ± 130.17	0.58
N %	0.64 ± 0.20	0.67 ± 0.17	0.61
L %	0.30 ± 0.19	0.29 ± 0.14	0.89
Hb (g/L)	100.77 ± 11.23	107.71 ± 11.06	0.04*
Hct (%)	30.51 ± 3.02	31.48 ± 2.28	0.32
CRP (mg/dl)	45.41 ± 33.47	40.24 ± 34.49	0.61
ESR (mm/hour)	61.09 ± 29.95	70.05 ± 20.73	0.45
CK-MB (U/L)	1.69 ± 1.57	1.19 ± 0.48	0.17
AST (U/L)	34.70 ± 11.91	37.43 ± 20.28	0.59
MIG/CXCL9 (ng/L)	1679.03 ± 197.03	514.21 ± 107.39	<0.01
TARC/CCL17(pg/ml)	953.74 ± 106.52	475.29 ± 67.05	<0.01
MIG/TARC	1.78 ± 0.26	1.09 ± 0.20	<0.01

*KD* Kawasaki disease, *Yr* year, *WBC* white blood cells counts, *Plt* platelets counts, *N%* percentage of neutrophils, *L%* percentage of leukomonocytes, *Hb* hemoglobin, *Hct* hematocrit, *CRP* C-reactive protein, *ESR* erythrocyte sedimentation rate, *CK-MB* creatine kinase-MB, *AST* aspartate aminotransferase, *MIG/CXCL9* Monokine induced by interferon-gamma, *TARC/CCL17* Thymus and activation-regulated chemokine, *MIG/TARC* the ratio of plasma MIG/CXCL9 and TARC/CCL17 levels; the *P* value < 0.05 is considered as statistically significant

### Correlations of MIG/CXCL9 and TARC/CCL17 with laboratory variables in all KD patients

**Fig. 1:**
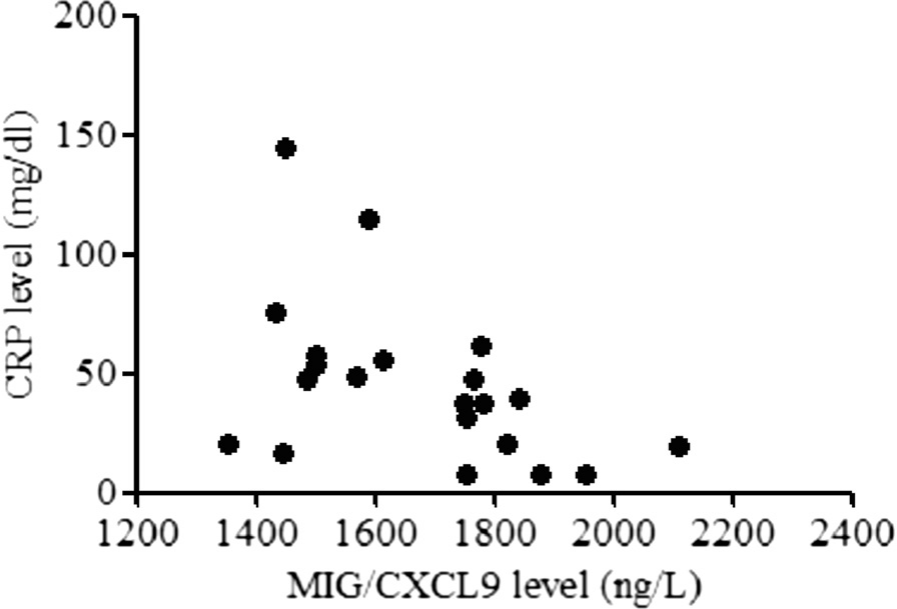
The correlations between plasma MIG/CXCL9 level and CRP level in KD-CAL group

**Fig. 2:**
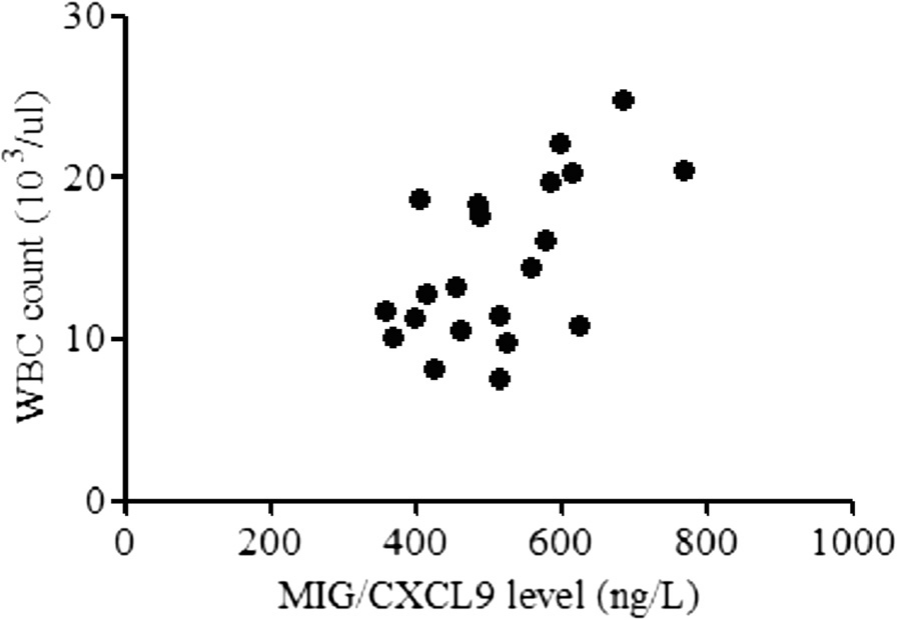
The correlations between plasma MIG/CXCL9 level and WBC count in KD-NCAL group

Plasma TARC/CCL17 levels in KD patients had no significant correlation with WBC, Plt, N%, Hb, Hct, CRP, ESR, AST, CK-MB (*P* > 0.05). However, plasma MIG/CXCL9 levels were negatively correlated with CRP (*r* = –0.464, *p* = 0.030) in KD-CAL group (Table 3, Fig. 1) and positively associated with WBC (*r* = 0.591, *p* = 0.005) in KD-NCAL group (Table 3, Fig. 2).

**Table 3:** Correlations of MIG/CXCL9 and TARC/CCL17 with laboratory variables in all KD patients

	KD-CAL (*n* = 22)				KD-NCAL (*n* = 21)			
	MIG/CXCL9		TARC/CCL17		MIG/CXCL9		TARC/CCL17	
	r	p	r	p	r	p	r	p
WBC (10^3^/ul)	–0.338	0.511	0.081	0.721	0.591	0.005*	0.412	0.064
Plt (10^3^/ul)	–0.263	0.719	0.018	0.938	–0.165	0.475	–0.205	0.374
N %	–0.025	0.915	0.113	0.625	–0.232	0.300	–0.372	0.089
L %	–0.013	0.955	–0.093	0.687	0.250	0.262	0.368	0.092
Hb (g/L)	0.297	0.180	0.050	0.826	0.089	0.700	0.102	0.661
Hct (%)	0.297	0.180	–0.048	0.833	0.039	0.868	0.092	0.691
CRP (mg/dl)	–0.464	0.030*	–0.241	0.279	0.283	0.214	0.399	0.074
ESR (mm/hour)	–0.369	0.091	0.145	0.520	0.153	0.507	–0.301	0.185
CK-MB (U/L)	–0.131	0.571	–0.007	0.977	0.091	0.687	–0.168	0.455
AST (U/L)	0.230	0.317	0.206	0.370	0.178	0.427	0.280	0.207

*KD* Kawasaki disease, *Yr* year, *WBC* white blood cells counts, *Plt* platelets counts, *N %* percentage of neutrophils, *L %* percentage of leukomonocytes, *Hb* hemoglobin, *Hct* hematocrit, *CRP* C-reactive protein, *ESR* erythrocyte sedimentation rate, *CK-MB* creatine kinase-MB, *AST* aspartate aminotransferase, *MIG/CXCL9* Monokine induced by interferon-gamma, *TARC/CCL17* Thymus and activation-regulated chemokine, *, *P*<0.05; the *P* value less than 0.05 is considered as statistically significant

## Discussion

In this study, we present a novel observation of the relationship between plasma MIG/CXCL9, TARC/CCL17 levels and coronary artery lesions in KD patients. We have found that KD patients had significantly higher plasma MIG/CXCL9 and TARC/CCL17 levels compared to the healthy controls. Furthermore, plasma MIG/CXCL9 and TARC/CCL17 levels were even higher in KD with CAL compared to KD without CAL. The increased levels of MIG/CXCL9 and TARC/CCL17 may suggest that these markers are involved in the development of coronary artery lesions. In addition, we have found that Hb is significantly decreased in KD with CAL, and plasma MIG/CXCL9 levels are negatively correlated with CRP in KD with CAL patients, whereas a positive correlation between plasma MIG/CXCL9 and WBC is observed in KD without CAL.

The etiology of KD remains unclear. Accumulated studies indicate that bacteria or virus infections are participated in the pathogenesis of KD [8, 9]. During the immunoreaction to infections, T cells were abnormally functioned. Matsubara [10] found that the numbers of CD4^+^ T cells were increased and the subsets of T cells were imbalanced. In Hahn’s study [11], T cells of KD were cultured *in vitro*, and as a result, levels of IFN-γ and IL-4, which were the representative factors of T helper cells, had no significant changes. Kimura [12] demonstrated that T cells were not activated and they were inhibited in the acute period of KD. So the data are controversial regarding the role and the balance of T cells in KD patients.

Chemotactic factors are a class of small molecular proteins that specifically collect and activate leukocytes. They are widely found in pathological processes such as inflammation, immunization, angiogenesis and hemopoiesis [13–16]. When inflammatory signals are active, chemotactic factors are enhanced. They can induce leukocytes and activate T cells migrating to inflammatory sites. Cytokines are secreted by T cells or macrophagocytes to control the inflammation. Plasma levels of chemotactic factors can represent the levels of activated T cells in inflammation. According to the sequences of cysteine in chemistry structure, chemotactic factors are divided into four different subfamilies: Cys-X-Cys (CXC), Cys-Cys (CC), Cys (C), Cys-X-3Cys (CX3C). MIG/CXCL9 belongs to the subfamily of CXC and TARC/CCL17 is part of CC subfamily. MIG/CXCL9 and TARC/CCL17 are chemoattractants for Th1 and Th2 cells and specifically activate Th1 and Th2 cells. They are up-regulated in inflammatory sites and constitutively expressed to induce the migration of Th1 and Th2 cells to control inflammation. So the plasma levels of MIG/CXCL9 and TARC/CCL17 can reflect the numbers of activated Th1 and Th2 cells in vasculitis sites in KD patients. Most reports studied cytokines levels like IFN-γ and IL- 4 secreted by Th1 and Th2 cells to evaluate the function of T cells in KD. In our study, we have tried another way by measuring plasma levels of MIG/CXCL9 and TARC/CCL17 in KD patients. In Lee CP’s report [5], plasma TARC levels were significantly higher in acute KD before IVIG treatment, but they found that plasma TARC/CCL17 levels of pre-IVIG, post-IVIG, and subacute stages did not differ significantly in the occurrence of CAL, coronary artery aneurysm (CAA), and the responsiveness in KD. Here, our study demonstrate that plasma levels of MIG/CXCL9 and TARC/CCL17 in patients with KD were significantly higher than those of healthy controls. Furthermore, the plasma levels of MIG/CXCL9 and TARC/CCL17 are even higher in KD-CAL group compared to those in KD-NCAL group (P < 0.05). Considering the chemotactic and proliferative effect of these molecules on Th1 and Th2 cells, the significant increasing of plasma levels of MIG/CXCL9 and TARC/CCL17 in KD patients should be an indication that Th1 and Th2 cells are activated and are positively associated with the development of immunoreaction in acute period of KD. T-cell activation of immunoreaction is identified to be the critical factor in determining susceptibility and severity of KD in children [17, 18]. Previous studies about super-antigen induced by immunoreaction of infections indicated that abnormal activation of T cells was the initial and key procedure of KD vascular lesions [3]. The significant increasing of plasma MIG/CXCL9 and TARC/CCL17 in KD with CAL could activate Th1 and Th2 cells and induce their stronger secretion of cytokines. Wang [19] found that Th1/Th2 cytokine profiles identified significantly increasing of cytokines in KD patients. We think that the stimulation of the cytokine cascade and the activation of endothelial cells plus the abnormal function of T cells could be the key events leading to coronary artery lesions in the acute phase of KD.

Since Th1 and Th2 cells can be induced by MIG/CXCL9 and TARC/CCL17 and migrate to vasculitis sites in KD, the levels of activated Th1 and Th2 cells should have an appropriate ratio and play a well-balanced role. T cells are found imbalanced in many autoimmune diseases. Kimura demonstrated that Th1 and Th2 cells in KD were well-balanced [12]. However, Matsubara found the balance of Th1/Th2 cells were altered [10]. We have compared the ratios of MIG/TARC to reveal the balance between Th1 and Th2 cells. The results have shown that the ratios of MIG/TARC in KD patients are significantly higher than those in healthy subjects. And furthermost, we have found that MIG/TARC ratios in KD with CAL show a significant difference compared to KD without CAL. These data suggested that when attacked by unidentified antigens in KD, both Th1and Th2 cells could be increased stimulated by MIG/CXCL9 and TARC/CCL17. However, the increasing speed of these T helper cells is not balanced and this imbalance might mediate immune lesions of vessels in KD patients.

Most previous studies indicated that the inflammatory indexes such as WBC, CRP and ESR were increased, and Hb, Plt, Hct were also changed in Kawasaki disease [20]. In our study, hemoglobin was decreased in KD with CAL. Although we don’t know the mechanism of hemoglobin changes for the moment, the present data suggest that the decreased levels of hemoglobin could be a higher risk factor for CAL in KD patients. A hypoxic environment is induced by the decrease of hemoglobin, which may accelerate inflammation and causing endothelial cells injury. In addition, plasma MIG/CXCL9 levels in KD patients with CAL had significantly negative correlation with CRP, whereas a positive correlation with WBC in KD without CAL. As we know, WBC and CRP are indexes of inflammation, high WBC and CRP indicate a serious level of inflammation. When inflammation occurs, MIG/CXCL9 and TARC/CCL17 can stimulate Th1 and Th2 cells secreting cytokines to regulate the immunoreaction during the development of vasculitis in KD, and so plasma MIG/CXCL9 levels increase with the development of a serious inflammation. However, when vasculitis develops into CAL, plasma MIG/CXCL9 levels could be negatively feedback regulated while TARC/CCL17 increase persistently. The different increasing speed of MIG/CXCL9 and TARC/CCL17 levels may result in an imbalance of Th1 and Th2 cells.

## Conclusion

Our study demonstrates that the Th1 and Th2 cells may be activated unevenly by plasma MIG/CXCL9 and TARC/CCL17 in acute period of KD. The imbalance of Th1 and Th2 cells may be a cause associated with the development of e immune lesions of vessels in KD patients. However, both the KD and healthy control cases in this study are limited, we should be cautious in interpret our data. More studies are needed with a large number of patients and carrying out more functional tests of T helper cells in KD patients.

## Abbreviations

AST: Aspartate aminotransferase
C: Cys
CAA: Coronary artery aneurysm
CAL: Coronary artery lesions
CC: Cys-Cys
CK-MB: Creatine kinase-MB
CRP: C-reactive protein
CXC: Cys-X-Cys
CX3C: Cys-X-3Cys
EDTA: Ethylene diamine tetraacetic acid
ELISA: Enzyme-linked immunosorbent assay
ESR: Erythrocyte sedimentation rate
Hb: Hemoglobin
Hct: Hematocrit
IFN-γ: Interferon-gamma
IL-4: Interleukin-4
KD: Kawasaki disease
L%: Percentage of leukomonocytes
MCLS: Mucocutaneous lymphnode syndrome
MIG/CXCL9: Monokine induced by interferon-gamma/Chemokine (C-X-C motif) ligand 9
N%: Percentage of neutrophils
NCAL: Non-CAL
Plt: Platelet
SD: Standard deviation
TARC/CCL17: Thymus and activation-regulated chemokine/Chemokine (C-C motif) ligand 17
Th1: T helper type-1
Th2: T helper type-2
WBC: White blood cellsl
